# Evaluation of Bacterial Strains as a Sustainable Approach for Control of *Myzus cerasi* (F.) (Hemiptera: Aphididae) Under Laboratory and Field Conditions

**DOI:** 10.3390/insects16080857

**Published:** 2025-08-18

**Authors:** Yeşim Bulak Korkmaz

**Affiliations:** Department of Plant Protection, Faculty of Agriculture, Atatürk University, Erzurum 25240, Türkiye; yesim.bulak@atauni.edu.tr

**Keywords:** *Bacillus* spp., biological control, biopesticides, cherry aphid, integrated pest management

## Abstract

This study aims to evaluate the insecticidal activity of four bacterial strains against *M. cerasi* under both laboratory and field conditions, in order to provide a more sustainable and eco-friendly alternative to chemical insecticides. Four environmentally friendly bacterial strains were evaluated for their potential as biocontrol agents: *Brevibacillus brevis* (FD-1), *Bacillus cereus* (FD-63), *Bacillus thuringiensis* subsp. *kenyae* (FDP-8), and *Bacillus thuringiensis* subsp. *kurstaki* (FDP-41). Experiments were conducted under both controlled laboratory and field conditions. Data showed that mortality rates ranged from 33.33% to 93.33% under controlled conditions and from 13.33% to 50% under field conditions over the 96 h period. *B. thuringiensis* subsp. *kurstaki* (FDP-41) and *B. thuringiensis* subsp. *kenyae* (FDP-8) were the most effective bacterial strains against the aphids in controlled conditions. The percentage of mortality related to these applications was 93.33% and 80% in 96 h under laboratory conditions. However, the effectiveness of *B. thuringiensis* subsp. *kurstaki* (FDP-41) was 50% at 96 h in the field. These findings suggest that *B. thuringiensis* subsp. *kurstaki* and *B. thuringiensis* subsp. *kenyae* hold significant potential as biological control agents against *M. cerasi*. Additionally, FDP-41 showed the lowest LT_50_ value of 25.37 h in the laboratory and 86.40 h in the field. This indicates a much more rapid and potent effect on *M. cerasi* compared to other strains. As a result, it was concluded that these bacterial strains may be used successfully as biocontrol agents in the biological control of *M. cerasi*. Their successful use could reduce reliance on synthetic insecticides, thereby minimizing pesticide residues on crops and contributing to safer agricultural production and environmental sustainability.

## Simple Summary

The cherry aphid, *Myzus cerasi*, is a harmful pest that damages cherry trees. Due to the detrimental effects of chemical pesticides on both environmental and human health, increasing emphasis has been placed on the development and implementation of safer and more sustainable pest management strategies. In this study, four bacterial species were tested as potential biological control agents against *M. cerasi*. The bacteria were applied in both laboratory and real field conditions. The results showed that two of the bacteria, *Bacillus thuringiensis* subsp. *kurstaki* and *Bacillus thuringiensis* subsp. *kenyae*, were highly effective in controlling the pest in the laboratory, with mortality rates reaching up to 93%. However, their effectiveness was lower in the field. These findings indicate that the tested *B. thuringiensis* strains, particularly FDP-41 and FDP-8, have the potential to be integrated into Integrated Pest Management (IPM) programs as biological control agents, offering a targeted and environmentally compatible alternative to chemical insecticides.

## 1. Introduction

The black cherry aphid, *Myzus cerasi* (Fabricius) (Hemiptera: Aphididae), is a cosmopolitan species that infests various plant families such as Brassicaceae, Plantaginaceae, Rosaceae, Rubiaceae, and Scrophulariaceae [1], and is particularly known as one of the major pests of cherry trees (*Prunus* spp.) [2]. This aphid causes deformation of the young leaves, impacts the photosynthesis rate, induces inwards curling and drying of the upper shoots, and causes the formation of fumagin on leaves and fruit of sweet cherries and sour cherries [3].

*Myzus cerasi* has been reported to potentially transmit several plant viruses under experimental conditions, although its role as a vector under natural field conditions remains unclear [4,5,6,7]. Serious damage to young trees are usually reported in nurseries [5,8,9]. *M. cerasi* develop a holocyclic and heteroecious life cycle, migrating between primary host (trees) and secondary hosts (herbaceous plants) throughout the year. Wingless females emerge in spring from eggs deposited during the winter at the bases of buds and young shoots of cherry trees, where they feed and develop on the young leaves. Subsequently, winged individuals migrate to secondary hosts in early summer [3,10,11]. Temperature, relative humidity, development rate, and the diversity and density of natural enemies affect aphid populations [12].

The management of black cherry aphids in commercial orchards has relied on repeated applications of broad-spectrum chemical insecticides [2]. While such insecticides can suppress *M. cerasi* populations in the short term, their intensive use has raised several problems. Aphids are prone to developing resistance under sustained chemical pressure, rendering some pesticides progressively less effective [13]. Moreover, the improper application of pesticides may adversely affect populations of natural enemies of aphids, such as ladybird beetles and parasitoids [2,13].

In recent years, biocontrol agents have emerged as a promising alternative to chemical pesticides in pest management. These environmentally friendly methods are gaining popularity due to their ability to minimize harm to non-target organisms and reduce chemical residues in ecosystems [14,15]. Among these biocontrol agents, bacterial species particularly those from the *Bacillus* genus have received increasing research for their biopesticidal properties, offering a sustainable and ecologically safe approach to pest control in agriculture [16,17,18].

*Bacillus thuringiensis* (Bt) is a ubiquitous, Gram-positive, rod-shaped, sporulating, and facultatively anaerobic bacterium. It has been isolated worldwide from a great diversity of ecosystems including soil, water, dead insects, leaves from deciduous trees and diverse conifers, as well as necrotic human tissues [19,20,21,22,23,24]. It is considered low risk because it specifically targets certain pests without affecting humans, animals, or beneficial insects, and for this reason, it is the most widely used species [13,25,26] as these bacteria synthesize parasporal crystalline inclusions that produce Cry and Cyt proteins. Some of these proteins are toxic to various insect orders. Numerous studies demonstrated that these toxins can be successfully utilized as bioinsecticides against aphids, caterpillars, beetles, mosquitoes, and blackflies [23,26,27,28,29,30,31]. Moreover, Bt has been shown to exhibit high toxicity against many sap-sucking insects of the order Hemiptera, primarily due to the production of vegetative insecticidal proteins (Vip). Vip proteins are secreted during the vegetative growth phase and have been demonstrated to act through a different mode of action, effectively targeting sap-feeding insects such as aphids [23,32,33,34]. Owing to these characteristics, Bt is used in both biotechnological and industrial areas [35,36,37,38].

In fact, *Bacillus thuringiensis* (Bt) and closely related *Bacillus* species account for approximately 95% of the global biopesticide market, due to their proven effectiveness and broad application as microbial control agents [39]. *B. thuringiensis* subsp. *kurstaki* and *B. thuringiensis* subsp. *kenyae* showed insecticidal activity against aphids [40].

This study hypothesizes that the bacterial strains *B. brevis* (FD-1), *B. cereus* (FD-63), Bt subsp. *kenyae* (FDP-8), and Bt subsp. *kurstaki* (FDP-41) will significantly reduce *M. cerasi* populations under both laboratory and field conditions. Furthermore, it is expected that some strains may exhibit insecticidal efficacy comparable to that of chemical insecticides, offering a more sustainable alternative for pest management. Accordingly, the aim of this study is to evaluate and compare the insecticidal efficacy of these bacterial strains against *M. cerasi* under controlled and field conditions.

## 2. Materials and Methods

### 2.1. Bacterial Strains Used in This Study

In this study, a total of four bacterial strains (FD-1: *Brevibacillus brevis* isolated from *Malacosoma neustria*, FD-63: *Bacillus cereus* isolated from *Yponomeuta evonymella*, FDP-8: *Bacillus thuringiensis* subsp. *kenyae* isolated from *Hypera postica*, FDP-41: *Bacillus thuringiensis* subsp. *kurstaki* isolated *Apion* spp.) were used to assess their insecticidal efficacy against *M. cerasi*. These strains were previously characterized for their entomopathogenic potential from insect carcass. (Table 1). They were obtained from the culture collection unit in the Plant Clinical Laboratory, Department of Plant Protection, Faculty of Agriculture at Atatürk University, Erzurum, Türkiye. The bacterial strains were kept at −86 °C in stock growth media containing 30% glycerol and Loria Broth (LB). These bacterial isolates were incubated on Tryptic Soy Agar (TSA; Oxoid Ltd., Basingstoke, Hampshire, UK) at 27 °C for 24 h. Following the incubation period, a single colony from each isolate was transferred into 500 mL Erlenmeyer flasks containing Tryptic Soy Broth (TSB; Oxoid Ltd., Basingstoke, Hampshire, UK) and cultured aerobically on a rotating shaker at 150 rpm for 48 h at 27 °C (Merck KGaA, Darmstadt, Germany). The resulting bacterial suspension was then diluted in sterile distilled water (sdH_2_O) to a final concentration of 1 × 10^8^ CFU mL^−1^ using a turbidimeter.

### 2.2. Laboratory Experiment

*Myzus cerasi* individuals were collected from Yakutiye, Erzurum, Türkiye (39°55′22.3″ N, 41°12′46.7″ E; altitude: 1850 m). The bacterial strains were cultured in three phases on nutrient agar (NA) medium at 30 °C for 24 h to obtain fresh colonies. One colony from each strain was inoculated into 300 mL of nutrient broth (NB) in separate Erlenmeyer flasks using sterile loops. The flasks were incubated in a thermostatic shaker at 250 rpm and 25 °C for 24 h. The bacterial density of the resulting aqueous culture was adjusted to 1 × 10^8^ CFU/mL using sterile NB medium, following and then transferred to sterile spray bottles. The insecticidal efficacy of the bacterial strains against *M. cerasi* was evaluated under controlled laboratory conditions (25 °C, 75–80% relative humidity, and a 16:8 h light: dark photoperiod). The cherry tree leaves naturally infested with *M. cerasi* were brought to a laboratory of the Plant Clinical Laboratory, Department of Plant Protection, Faculty of Agriculture at Atatürk University. Leaf area measurements were conducted to ensure that the selected leaves had similar surface areas, thereby maintaining consistency across experimental units. Each leaf was then placed in a Petri dish, and ten live *M. cerasi* individuals were transferred onto each leaf prior to treatment. Subsequently, a bacterial suspension of 1 × 10^8^ CFU/mL was sprayed onto the infested leaves. Control groups included leaves treated with sterile distilled water (negative control) and leaves treated with Acetamiprid (20% SP) (Agrobest Grup, İzmir, Türkiye). The experiment was conducted with three replicates. The number of dead aphids on the leaves was recorded after 24, 48, 72, and 96 h.

### 2.3. Field Experiment

The field experiments were conducted in a noncommercial fruit orchard located in Erzurum, Türkiye. The total orchard area was 1.8 decares (0.18 hectares), and the trees were six years old. No insecticides or other pest control managements were applied during the trial period, except for the bacterial treatments evaluated in this study. Shoots, each containing 12 leaves, were selected from various parts of a cherry tree infested with *M. cerasi.* The experiment was conducted with three replicates. Each leaf was sprayed with 1.5 mL of bacterial suspension (1 × 10^8^ CFU/mL) from a distance of 15 cm using a hand sprayer. For comparison, positive and negative control leaves were treated with Acetamiprid (20% SP) and sterile distilled water, respectively. All treated shoots were placed in tulle mesh cages measuring 45 × 45 cm and labeled according to their respective treatment groups. At 24 h after the initial application, 3 of the 12 leaves were randomly selected from each shoot and brought to the laboratory. The surface area of each leaf was measured using millimetric graph paper by carefully tracing the leaf outline and calculating the area in cm^2^. These measurements confirmed that the selected leaves had comparable surface areas. Subsequently, the numbers of live and dead *M. cerasi* individuals were recorded under a stereomicroscope (Leica EZ4, Leica Microsystems, Wetzlar, Germany). The mortality rate (%) was calculated independently for each leaf using the following formula: Mortality (%) = (Number of Dead Aphids/Total Number of Aphids) × 100. The resulting mortality percentages were used in statistical analysis to evaluate the efficacy of each bacterial strain. This process was repeated at the 48th, 72nd, and 96th hours by taking the remaining leaves.

### 2.4. Statistical Analysis

Prior to statistical analysis, the raw counts were converted into mortality percentages (%). All data were analyzed using SPSS version 27.0 (IBM Corp., Armonk, NY, USA) statistical software. A repeated-measures ANOVA (General Linear Model procedure) was used with Time (24, 48, 72, 96 h) as the within-subjects factor and Treatment (bacterial strain) as the between-subjects factor to test the main effects and their interaction on cumulative mortality. Pairwise comparisons among treatments at each time point were performed using Tukey’s HSD only when the ANOVA indicated significant differences. Statistical significance was determined at the *p* ≤ 0.05 level. Additionally, the LT_50_ values of bacterial strains, along with their 95% confidence limits, were estimated through probit analysis.

## 3. Results

Exposure time and bacterial strain significantly affected the cumulative mortality of *M. cerasi* under both laboratory and field conditions. Mortality patterns exhibited distinct temporal dynamics and varied notably among treatments, with these differences being evident across both experimental environments. Comprehensive statistical outcomes are presented in Table 2 and Table 3.

### 3.1. Effectiveness of FDP-41

**Figure 1:**
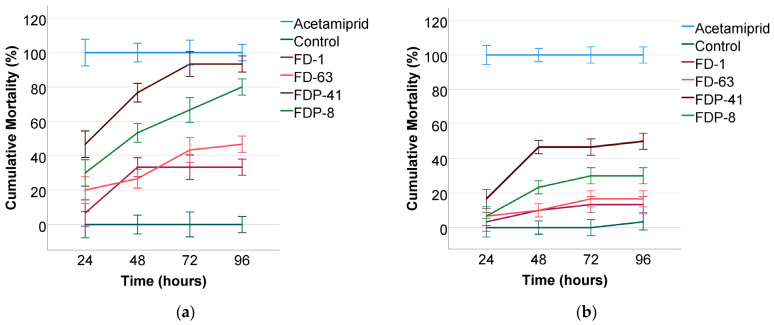
Cumulative mortality rates (%) of *M. cerasi* at 24, 48, 72, and 96 h after exposure to different bacterial strains (FD-1, FD-63, FDP-8, FDP-41), a chemical insecticide (Acetamiprid), and a control treatment (**a**) under laboratory conditions and (**b**) under field conditions. Error bars represent the standard error of the mean (±SE).

FDP-41 demonstrated the highest insecticidal efficacy against *M. cerasi* among the tested strains. Under laboratory conditions, mortality rates increased significantly over time: 46.67% at 24 h, 76.67% at 48 h, and 93.33% at both 72 and 96 h. This increasing trend reached a plateau after 72 h, indicating rapid and sustained effectiveness (Figure 1a). The differences over time were statistically significant (*p* < 0.001). Under field conditions, mortality rates were lower yet still substantial: 16.67%, 46.67%, 46.67%, and 50% at 24, 48, 72, and 96 h, respectively. Despite a clear reduction in efficacy compared to laboratory results, the effects remained significant (*p* < 0.001), confirming FDP-41’s potential under natural conditions, albeit with some environmental constraints. Across all observation times, the mean mortality rate of FDP-41 was 77.50% in the laboratory and 40% in the field, further highlighting its overall superior performance compared to other bacterial strains. Among the tested strains, FDP-8 exhibited the closest level of efficacy to FDP-41 (Figure 1b). However, in the laboratory, FDP-41 reached 93.33% mortality by 72 h, while FDP-8 achieved only 80% by 96 h. Under field conditions, FDP-41 attained 50% mortality, whereas FDP-8 plateaued at 30% with no further increase after 48 h. These findings indicate that FDP-41 is superior to FDP-8 in terms of both speed and consistency of insecticidal efficacy across different environments. In the laboratory, the Treatment factor showed a very high F-value (F = 600.937, *p* < 0.001), indicating large and significant differences in mortality among the bacterial strains. This confirms clear and strong variation in their insecticidal performance (Table 2). FDP-41 exhibited the most rapid insecticidal activity against *M. cerasi*, with LT_50_ = 25.37 ± 0.267 h in laboratory and LT_50_ = 86.40 ± 0.326 h in field. The corresponding mean survival times (MST) were also the shortest among all treatments (MST: 32.8 ± 1.2 h in the laboratory and 95.1 ± 1.9 h in the field) confirming its fast-acting and highly potent effect under both conditions (Table 3). This suggests that FDP-41 may be a promising candidate for practical field applications as a biological control agent. When compared to the other bacterial strains tested in this study, FDP-41 consistently outperformed in terms of both mortality rate and lethal time. While FDP-8 also showed insecticidal activity, particularly under laboratory conditions, it was LT_50_ = 46.969 ± 0.304 h, nearly twice as long as FDP-41, thus indicating a slower onset of mortality. FD-63 (LT_50_ = 87.758 ± 0.397 h) and FD-1 (LT_50_ = 102.476 ± 0.438 h) displayed moderate to low efficacy (Table 3). These comparative results reinforce the performance of FDP-41 and support its prioritization for further development and integration into sustainable pest management strategies.

### 3.2. Effectiveness of FDP-8

FDP-8 also exhibited strong insecticidal activity under laboratory conditions, with mortality rates rising from 30% at 24 h to 80% at 96 h. This trend corresponded to an overall mean mortality of 57.50% across all time points in the laboratory, indicating a consistent and substantial effect. Statistical analysis revealed a significant increase over time (*p* < 0.001). However, field performance was noticeably reduced (Figure 1b). Mortality rates reached only 6.67% at 24 h and plateaued after 48 h, with no increase beyond 30% by 96 h and yielding an overall mean mortality of 22.50%**.** Despite the lower values, the treatment effects were statistically significant (*p* < 0.001), though environmental factors likely constrained efficacy. The decrease in efficacy in field conditions may be due to fluctuating environmental parameters such as temperature change, which may disrupt bacterial viability or reduce aphid contact with the biopesticide. FDP-8 exhibited significantly higher insecticidal efficacy than FD-63 under both laboratory and field conditions (Figure 1a,b). In laboratory trials, FDP-8 reached a mortality rate of 80% within 96 h, whereas FD-63 achieved approximately half of this value over the same period. Differences in field performance were even more pronounced; FDP-8 maintained a consistent increase in mortality, reaching 30% by 96 h, while FD-63 plateaued at just 16.67% at 96 h. These differences were also statistically significant. FDP-8 demonstrate a considerable lethal time against *M. cerasi*, with LT_50_ = 46.969 ± 0.304 h under laboratory and LT_50_ = 138.38 ± 0.395 h under field conditions. MST were 60.2 ± 1.8 h and 150.7 ± 2.4 h, respectively (Table 3), indicating that while FDP-8 was more effective under controlled laboratory conditions, its activity was slower in the field (Figure 1a,b). While its activity was less rapid than that of FDP-41, it still outperformed FD-63 and FD-1 in both environments, suggesting its potential as an effective biological control option, particularly under controlled conditions.

### 3.3. Effectiveness of FD-63

FD-63 displayed moderate efficacy under laboratory conditions, with mortality increasing from 20% at 24 h to 46.67% at 96 h, corresponding to an overall mean mortality of 34.17% across all time points in the laboratory. Although less potent than FDP-41 and FDP-8 strains, the time-dependent trend was statistically significant. In contrast, in field conditions, FD-63 demonstrated mortality rates of 6.67%, 10%, 16.67%, and 16.67% at 24, 48, 72, and 96 h, respectively. The overall effectiveness of FD-63 under field conditions appeared relatively low, with mortality rates stabilizing at 16.67% after 72 h. FD-63 consistently induced higher cumulative mortality rates than FD-1 across both test environments (Figure 1a,b). In laboratory assays, FD-63 reached 46.67% mortality by 96 h, while FD-1 achieved 33.33%, with no further increase after 48 h. Under field conditions, FD-63 maintained a slight advantage, attaining 16.67% mortality compared to FD-1’s 13.33%. Although FD-63 demonstrated a relatively greater insecticidal impact than FD-1 within the tested timeframe, its overall efficacy remained clearly lower compared to FDP-41 and FDP-8. Under laboratory conditions, FD-63 exhibited an LT_50_ of 87.758 ± 0.397 h and an MST of 115.3 ± 2.3 h, indicating slower insecticidal activity compared with FDP-8 (46.969 ± 0.304 h; MST = 60.2 ± 1.8 h) and FDP-41 (25.37 ± 0.267 h; MST = 32.8 ± 1.2 h), but faster than FD-1 (102.476 ± 0.438 h; MST = 130.5 ± 2.5 h). Under field conditions, the LT_50_ for FD-63 increased to 178.454 ± 0.523 h, with an MST of 195.6 ± 2.9 h, reflecting a marked reduction in speed of action compared with laboratory results (Table 3).

### 3.4. Effectiveness of FD-1

FD-1 showed the lowest efficacy among the strains tested. Under laboratory conditions, mortality rose sharply between 24 and 48 h (6.67% to 33.33%) and remained constant thereafter, corresponding to an overall mean mortality of 26.67% across all time points in the laboratory. This stabilization indicates limited but consistent lethality. The treatment was statistically significant (*p* < 0.001). Field trials, however, resulted in minimal mortality (3.33% to 13.33%), yielding an overall mean mortality of 10% in the field. These results suggest poor field adaptability and low persistence for FD-1. FD-1 consistently exhibited the lowest efficacy among all tested strains (Figure 1a,b). While it showed a brief increase in mortality under laboratory conditions, the effect quickly plateaued and remained limited. In both laboratory and field trials, FD-1 was clearly less effective than FDP-41, FDP-8, and FD-63, indicating limited biocontrol potential under the current conditions. Under laboratory conditions, FD-1 showed an LT_50_ = 102.476 ± 0.438 h, and the highest MST (130.5 ± 2.5 h) among all strains, indicating the slowest insecticidal activity. Similarly, under field conditions, FD-1 had an LT_50_ = 192.135 ± 0.576 h and the highest MST (210.4 ± 3.1 h), confirming its relatively delayed effect compared with the other treatments (Table 3).

The comparative analysis of all tested bacterial strains, supported by repeated measurements, LT_50_, and MST estimates, revealed statistically significant differences in insecticidal performance against *M. cerasi* under both laboratory and field conditions. The clear superiority of Bt subspecies, particularly FDP-41, suggests that certain strains possess enhanced virulence and environmental resilience. However, the overall decline in field efficacy highlights the complex interaction between microbial agents and environmental variables. These findings provide a foundation for further interpretation regarding the practical implications of using bacterial biopesticides for *M. cerasi*’s control in integrated pest management systems.

## 4. Discussion

The present study demonstrated that all four bacterial strains tested exhibited insecticidal activity against *M. cerasi* under both laboratory and field conditions. However, their efficacy varied considerably depending on the bacterial species and environmental conditions. For all strains, mortality rates recorded under laboratory settings were significantly higher than those observed in the field, suggesting that environmental factors had a substantial influence on the bio efficacy of the tested biopesticides. Among the strains, FDP-41 achieved the highest and most consistent mortality across all time points in both environments. In contrast, FD-1 exhibited the lowest efficacy, particularly under field conditions. FDP-41 induced the highest mortality across all time points, reaching 93.33% at 96 h under laboratory conditions and 50% by 96 h in the field. These outcomes were supported by the lowest LT_50_ values observed in the study (LT_50_ = 25.37 h in the lab; LT_50_ = 86.40 h in the field), indicating a rapid and sustained insecticidal action. Similarly, other researchers evaluated the effects of various entomopathogenic bacterial strains isolated from citrus cultivated soils on *Hyalopterus pruni*, and reported that certain Bt strains caused mortality rates of up to 96% within 72 h [39]. In another study, laboratory results showed that Bt subsp. *kenyae* and *B. cereus* were the most effective strains against *Aphis pomi*, achieving mortality rates of 90% and 83.3%, respectively [44]. The high performance of FDP-41 may be attributed to its production of Cry and Cyt protein toxins, which are well known for their potent insecticidal activity against soft-bodied insects such as aphids [31,35,41], or it may be due to the presence of vegetative insecticidal proteins (Vip), which are known to be toxic in controlled conditions to many sap-sucking insects of the order Hemiptera [23,32,33,34]. Previous studies have confirmed the broad-spectrum activity of Bt subspecies, including their effectiveness against aphids and other sap-sucking pests [40,45,46,47,48].

FDP-8 also showed promising results, particularly under laboratory conditions, where it reached 80% mortality by 96 h (LT_50_ = 46.97 h). Similar findings were reported by Tozlu et al., who demonstrated that Bt subsp. *kurstaki* and Bt subsp. *kenyae* caused 100% mortality of *Tinnocallis saltans* within 48 h under laboratory conditions [40]. Consistent with our findings, a study conducted in Korea demonstrated that Bt strain AH-2 induced a mortality rate of up to 73% in *Aphis gossypii* within 96 h under laboratory conditions [47]. However, its field efficacy was notably lower, with a maximum mortality of 30% at 96 h (LT_50_ = 138.38 h), possibly due to environmental stressors such as ultraviolet (UV) radiation, fluctuating temperatures, and reduced contact between aphids and the bacterial formulation [49,50,51]. Furthermore, aphid population dynamics are known to be sensitive to temperature fluctuations. Average increase in temperature by 2 °C has been shown to increase the number of generations of aphids from 18 to 23 per year [52]. Although from the same genus, FDP-41 performed better than FDP-8, showing that subspecies differences affect efficacy.

On the other hand, FD-63 and FD-1 showed only moderate to low efficacy, especially under field conditions. While both strains have previously demonstrated activity against mosquito larvae and other insect species [16,43], their limited performance against *M. cerasi* may be due to host-specific physiological resistance or lower ingestion rates on the leaf surface.

The time-dependent increase in aphid mortality observed with FDP-41 and FDP-8 under laboratory conditions supports the hypothesis that longer exposure enhances the effectiveness of bacterial toxins. This pattern was less evident in FD-63 and FD-1, which showed a plateau in efficacy after 48 h (Figure 1). These results highlight the importance of exposure duration and pathogen virulence in determining the success of biocontrol agents. A study evaluated 40 different strains of Bt against *Myzus persicae* and reported mortality rates exceeding 70%, even at low concentrations of 10 ng/μL. Symptoms of infection appeared within 24 h, with peak effects observed at 72 h [13]. Similar findings have shown that prolonged exposure significantly enhances aphid mortality when treated with microbial insecticides, highlighting the importance of contact duration for biopesticide effectiveness [53].

Although the chemical insecticide acetamiprid produced 100% mortality under both laboratory and field conditions, it is associated with environmental concerns, including toxicity to non-target organisms and the development of insect resistance [48,54]. In contrast, biopesticides based on *Bacillus* spp. are regarded as environmentally safe alternatives, offering a more sustainable approach to aphid control in integrated pest management (IPM) programs [31,49,50,55]. However, their reduced field efficacy indicates the need for formulation improvements, such as protective carriers or additives that enhance persistence under environmental stress.

In conclusion, all four bacterial strains tested in this study exhibited insecticidal activity against *M. cerasi* under both laboratory and field conditions. However, their efficacy varied significantly depending on the strain and environmental conditions. FDP-41 emerged as the most effective strain, achieving the highest and most rapid mortality rates across both settings. FDP-8 also showed strong performance under laboratory conditions. However, its decreased field activity could be due to environmental stressors such as UV radiation and temperature fluctuations. These environmental factors may also have influenced the reduced efficacy observed in FD-63 and FD-1 under field conditions. These findings underscore the potential of FDP-41and FDP-8 as promising candidates for incorporation into Integrated Pest Management (IPM) programs targeting *M. cerasi*. Nevertheless, the observed drop in performance under field conditions highlights the importance of developing improved formulations that enhance environmental stability, bacterial viability, and persistence on plant surfaces.

Future research should focus on the development of microencapsulated formulations, UV-protective additives, oil-based or adhesive carriers, and biofilm-enhancing compounds to improve field performance. Additionally, genetic characterization, toxin profiling, and in-depth understanding of the mechanism of action of these strains will be essential for guiding product development. As a result, such efforts will be essential to establish sustainable and effective biological control strategies for aphid management in cherry orchards and potentially other horticultural crops.

## Figures and Tables

**Table 1:** Bacterial strains used in this study.

Bacterial Strains No	Isolated from	MIS Identification Results	Reference
FDP-41	*Apion* spp.	*Bacillus thuringiensis* subsp. *kurstaki*	[41]
FDP-8	*Hypera postica*	*Bacillus thuringiensis* subsp. *kenyae*	[41]
FD-1	*Malacosoma neustria*	*Brevibacillus brevis*	[42]
FD-63	*Yponomeuta evonymella*	*Bacillus cereus*	[43]

**Table 2:** Summary of the effects of Time (24, 48, 72, and 96 h), Treatment (bacterial strain), and their interaction (Time × Treatment) on *M. cerasi* mortality under laboratory and field conditions.

Condition	Source of Variation	F	*df*	*p*-Value
Laboratory	Time	49.207	3, 8	<0.001
	Treatment	600.937	5, 12	<0.001
	Time × Treatment	8.738	15, 36	<0.001
Field	Time	152.594	3, 8	<0.001
	Treatment	21.419	5, 12	<0.001
	Time × Treatment	203.804	15, 36	<0.001

All main effects and interactions were statistically significant (*p* < 0.001).

**Table 3:** LT_50_ ± SE and Mean Survival Time (MST) ± SE of four bacterial strains tested against *M. cerasi* under laboratory and field conditions.

Treatments	Laboratory		Field	
	LT_50_ ± SE	MST ± SE	LT_50_ ± SE	MST ± SE
FD-1	102.476 ± 0.438	130.5 ± 2.5	192.135 ± 0.576	210.4 ± 3.1
FD-63	87.758 ± 0.397	115.3 ± 2.3	178.454 ± 0.523	195.6 ± 2.9
FDP-8	46.969 ± 0.304	60.2 ± 1.8	138.384 ± 0.395	150.7 ± 2.4
FDP-41	25.37 ± 0.267	32.8 ± 1.2	86.404 ± 0.326	95.1 ± 1.9

## Data Availability

The original contributions presented in this study are included in the article. Further inquiries can be directed to the corresponding author.

## References

[1] Holman J. (2009). Host Plant Catalog of Aphids, Palearctic Region.

[2] McLaren G.F., Fraser J.A. (2002). Autumn and Spring Control of Black Cherry Aphid on Sweet Cherry in Central Otago. N. Z. Plant Prot..

[3] Kök Ş., Kasap İ. (2024). Seasonal population fluctuation and life history in different temperatures of *Myzus cerasi* (Hemiptera: Aphididae) on cherry trees: A field and laboratory study. J. Econ. Entomol..

[4] Bokx J.A., Piron P.G.M. (1990). Relative efficiency of a number of aphid species in the transmission of potato virus YN in the Netherlands. Neth. J. Plant Pathol..

[5] Blackman R.L., Eastop V.F. (2000). Aphids on the World’s Crops: An Identification and Information Guide.

[6] Basky Z., Almási A. (2005). Differences in aphid transmissibility and translocation between PVYN and PVYO isolates. J. Pest Sci..

[7] Nanayakkara U.N., Nie X., Giguère M., Zhang J., Boquel S., Pelletier Y. (2012). Aphid feeding behavior in relation to potato virus Y (PVY) acquisition. J. Econ. Entomol..

[8] Bielicki P., Badowska-Czubik T., Rozpara E. (2011). Pests occurring in plum organic nursery. J. Res. Appl. Agric. Eng..

[9] Wojciechowicz-Żytko E., Dobińska-Graczyk M. (2025). Urban green space as a reservoir of predatory syrphids (Diptera, Syrphidae) for aphid control in cities. Agronomy.

[10] Alford D.V. (2012). Pests of Ornamental Trees, Shrubs and Flowers. A Colour Handbook.

[11] Alford D.V. (2014). Pests of Fruit Crops. A Colour Handbook.

[12] Kindlmann P., Jarošík V., Dixon A.F.G., van Emden H.F., Harrington R. (2007). Population dynamics. Aphids as Crop Pests.

[13] Torres-Quintero M.C., Peña-Chora G., Hernández-Velázquez V.M., Arenas-Sosa I. (2015). Signs of *Bacillus thuringiensis* (Bacillales: Bacillaceae) infection in *Myzus persicae* (Hemiptera: Aphididae): Koch’s postulates. Fla. Entomol..

[14] Kumar P., Kamle M., Borah R., Mahato D.K., Sharma B. (2021). *Bacillus thuringiensis* as microbial biopesticide: Uses and application for sustainable agriculture. Egypt. J. Biol. Pest Control..

[15] Gomis-Cebolla J., Berry C. (2023). *Bacillus thuringiensis* as a biofertilizer in crops and their implications in the control of phytopathogens and insect pests. Pest Manag. Sci..

[16] Dadaşoğlu F., Karagöz K., Kotan R., Sarıhan F., Yıldırım E., Saraç S., Harmantepe F. (2013). Biolarvicidal effects of nine *Bacillus* strains against larvae of *Culex pipiens* Linnaeus, 1758 (Diptera: Culicidae) and nontarget organisms. Egypt. J. Biol. Pest Control..

[17] Dadaşoğlu F., Tozlu G., Kotan R., Göktürk T., Karagöz K. (2016). Biological control of pine sawfly (*Diprion pini* L.) and molecular characterization of effective strains. Rom. Biotechnol. Lett..

[18] Mampallil L.J., Faizal M.H., Anith K.N. (2017). Bacterial bioagents for insect pest management. J. Entomol. Zool. Stud..

[19] Höfte H., Whiteley H.R. (1989). Insecticidal crystal proteins of *Bacillus thuringiensis*. Microbiol. Rev..

[20] Knowles B.H., Dow J.A.T. (1993). The crystal delta-endotoxins of *Bacillus thuringiensis*—Models for their mechanism of action on the insect gut. BioEssays.

[21] Raymond B., Johnston P.R., Nielsen-LeRoux C., Lereclus D., Crickmore N. (2010). *Bacillus thuringiensis*: An impotent pathogen?. Trends Microbiol..

[22] Roh J.Y., Choi J.Y., Li M.S., Jin B.R., Je Y.H. (2007). *Bacillus thuringiensis* as a specific, safe, and effective tool for insect pest control. J. Mol. Biol..

[23] Palma L., Muñoz D., Berry C., Murillo J., Caballero P. (2014). *Bacillus thuringiensis* toxins: An overview of their biocidal activity. Toxins.

[24] Toledo-Hernández E., Torres-Quíntero M.C., Mancilla-Dorantes I., Sotelo-Leyva C., Delgado-Núñez E.J., Hernández-Velázquez V.M., Dunstand-Guzmán E., Salinas-Sánchez D.O., Peña-Chora G. (2025). Entomopathogenic bacteria species and toxins targeting aphids (Hemiptera: Aphididae): A review. Plants.

[25] 25. Konecka E., Kaznowski A., Grzesiek W., Nowicki P., Czarniewska E., Baranek J. (2020). Synergistic interaction between carvacrol and *Bacillus thuringiensis* crystalline proteins against *Cydia pomonella* and *Spodoptera exigua*. BioControl.

[26] 26. Baranek J., Pluskota M., Rusin M., Konecka E., Kaznowski A., Wiland-Szymańska J. (2023). Insecticidal activity of *Bacillus thuringiensis* strains isolated from tropical greenhouses towards *Cydia pomonella* and *Spodoptera exigua* larvae. BioControl.

[27] Alper M., Güneş H., Civelek H., Dursun O., Eskin A. (2014). Toxic effects of some native *Bacillus thuringiensis* Berliner (Bacillales: Bacillaceae) isolates against *Tetranychus urticae* Koch (Acarina: Tetranychidae), *Ceroplastes rusci* L. (Homoptera: Coccidae) and *Ceratitis capitata* (Wiedemann) (Diptera: Tephritidae). Bull. Entomol. Soc. Turk..

[28] Güneş H., Alper M., Çöl B., Tunca H. (2016). Bioactivities of cry gene positive *Bacillus thuringiensis* (Berliner) (Bacillales: Bacillaceae) strains on *Ephestia kuehniella* Zeller, 1879 and *Plodia interpunctella* (Hübner, 1813) (Lepidoptera: Pyralidae). Turk. J. Entomol..

[29] Şahin B., Gomis-Cebolla J., Güneş H., Ferré J. (2018). Characterization of *Bacillus thuringiensis* isolates by their insecticidal activity and their production of Cry and Vip3 proteins. PLoS ONE.

[30] Crespo A.L., Spencer T.A., Nekl E., Pusztai-Carey M., Moar W.J., Siegfried B.D. (2008). Comparison and validation of methods to quantify Cry1Ab toxin from *Bacillus thuringiensis* for standardization of insect bioassays. Appl. Environ. Microbiol..

[31] Palma L., Muñoz D., Berry C., Murillo J., De Escudero I.R., Caballero P. (2014). Molecular and insecticidal characterization of a novel Cry-related protein from *Bacillus thuringiensis* toxic against *Myzus persicae*. Toxins.

[32] Sattar S., Maiti M.K. (2011). Molecular characterization of a novel vegetative insecticidal protein from *Bacillus thuringiensis* effective against sap-sucking insect pest. J. Microbiol. Biotechnol..

[33] Bel Y., Ferré J., Hernández-Martínez P. (2020). *Bacillus thuringiensis* toxins: Functional characterization and mechanism of action. Toxins.

[34] Gupta M., Kumar H., Kaur S. (2021). Vegetative insecticidal protein (Vip): A potential contender from *Bacillus thuringiensis* for efficient management of various detrimental agricultural pests. Front. Microbiol..

[35] Schnepf E., Crickmore N., Van Rie J., Lereclus D., Baum J., Feitelson J., Zeigler D.R., Dean D. (1998). *Bacillus thuringiensis* and its pesticidal crystal proteins. Microbiol. Mol. Biol. Rev..

[36] Rooney A.P., Price N.P., Ehrhardt C., Swezey J.L., Bannan J.D. (2009). Phylogeny and molecular taxonomy of the *Bacillus subtilis* species complex and description of *Bacillus subtilis* subsp. *inaquosorum* subsp. nov. Int. J. Syst. Evol. Microbiol..

[37] Vovo A., Martínez de Castro D., Sánchez J., Cantón P.E., Mendoza G., Gómez I., Onofre J., Ocelotl J., Soberón M., Alouf J., Ladant D., Popoff M. (2015). Mechanism of action of *Bacillus thuringiensis* insecticidal toxins and their use in the control of insect pests. The Comprehensive Sourcebook of Bacterial Protein Toxins.

[38] Pomari E., Orza P., Bernardi M., Fracchetti F., Campedelli I., De Marta P., Recchia A., Paradies P., Buonfrate D. (2024). A pilot study for the characterization of *Bacillus* spp. and analysis of possible *B. thuringiensis*-*Strongyloides stercoralis* correlation. Microorganisms.

[39] Hafsa M., Benfekih L.A. (2024). New insights on entomopathogenic bacteria isolated from soil of citrus crops to combat the polyphagous aphid pest *Hyalopterus pruni* (Geoffroy 1762) (Hemiptera: Aphididae). Egypt. J. Biol. Pest Control..

[40] Tozlu E., Tozlu G., Kotan R., Çalmaşur Ö., Dadaşoğlu F. (2021). Investigation of some entomopathogens as biocontrol agents of *Tinocallis (Sappocallis) saltans* (Nevsky, 1929) (Hemiptera: Aphididae). Turk. J. Agric. For..

[41] Tozlu E., Dadaşoğlu F., Kotan R., Tozlu G. (2011). Insecticidal effect of some bacteria on *Bruchus dentipes* Baudi (Coleoptera: Bruchidae). Fresenius Environ. Bull..

[42] Göktürk T., Tozlu E., Kotan R. (2018). Prospects of entomopathogenic bacteria and fungi for biological control of *Ricania simulans* (Walker, 1851) (Hemiptera: Ricaniidae). Pakistan J. Zool..

[43] Tozlu E., Tozlu G., Kotan R., Tekiner N., Dadaşoğlu F., Göktürk T. (2022). Eco-friendly control method against invasive pest box tree moth (*Cydalima perspectalis* (Walker) (Lepidoptera: Crambidae)). Egypt. J. Biol. Pest Control..

[44] Narmanlıoglu H.K., Dadaşoğlu F. (2021). Investigation of the possibilities use of some bacterial biopesticides in the biological control against *Aphis pomi* (De Geer, 1773). Fresenius Enviromental Bull..

[45] Bravo A., Soberón M. (2008). How to cope with insect resistance to Bt toxins?. Trends Biotechnol..

[46] Alahyane H., Ouknin M., Alahyane A., Aboussaid H., Oufdou K., El Messoussi S., Majidi L. (2021). Aphicidal activities of Moroccan *Bacillus thuringiensis* strains against cotton aphid (*Aphis gossypii*). Biointerface Res. Appl. Chem..

[47] Ajuna H.B., Kim I., Han Y.S., Maung C.E.H., Kim K.Y. (2021). Aphicidal activity of *Bacillus thuringiensis* strain AH-2 against cotton aphid (*Aphis gossypii*). Entomol. Res..

[48] Wu K., Guo Y. (2003). Influences of *Bacillus thuringiensis* Berliner cotton planting on population dynamics of the cotton aphid, *Aphis gossypii* Glover, in northern China. Environ. Entomol..

[49] Ling L.G., Menn J.J. (2000). Biopesticides: A review of their action, applications and efficacy. Pest Manag. Sci..

[50] Jeschke P., Nauen R., Schindler M., Elbert A. (2011). Overview of the status and global strategy for neonicotinoids. J. Agric. Food Chem..

[51] Palma L., de Escudero I.R., Maneru-Oria F., Berry C., Caballero P. (2024). UV protection and insecticidal activity of micro-encapsulated Vip3Ag4 protein in *Bacillus megaterium*. Toxicon.

[52] Harrington R., Clark S., Kindlmann P., Dixon A.F.G., Michaud J.P. (2010). Trends in the timings of the start and end of annual flight periods. Aphid Biodiversity under Environmental Change: Patterns and Processes.

[53] Fernández-Grandon G.M., Harte S.J., Ewany J., Bray D., Stevenson P.C. (2020). Additive effect of botanical insecticide and entomopathogenic fungi on pest mortality and the behavioral response of its natural enemy. Plants.

[54] Bravo A., Gill S.S., Soberón M. (2007). Mode of action of *Bacillus thuringiensis* Cry and Cyt toxins and their potential for insect control. Toxicon.

[55] Ortiz A., Sansinenea E. (2023). Genetically modified plants based on *Bacillus* genes and commercial *Bacillus*-based biopesticides for sustainable agriculture. Horticulturae.

